# Mental Health Status of Adults with Cardiovascular or Metabolic Diseases by Gender

**DOI:** 10.3390/ijerph18020514

**Published:** 2021-01-10

**Authors:** Yeunhee Kwak, Yoonjung Kim, Soo Jin Kwon, Haekyung Chung

**Affiliations:** 1 Red Cross College of Nursing, Chung-Ang University, Seoul 06974, Korea; kwak0613@cau.ac.kr (Y.K.); kyung1104@cau.ac.kr (H.C.); 2 Department of Nursing, Ansan University, Ansan 15328, Korea; soojinyk@gmail.com

**Keywords:** cardiovascular diseases, gender, mental health, metabolic diseases

## Abstract

This study aimed to compare mental health in people with cardiovascular or metabolic diseases and the general adult population in each gender. Cardiovascular and metabolic diseases affect mental health, and the prognosis and incidence rates of these diseases differ by age. To date, studies comparing mental health in adults with cardiovascular or metabolic diseases by gender and with the general population have been insufficient. This work is a cross-sectional study. Data from 9828 men and 13,389 women aged 19 years or older from the Korea National Health and Nutrition Examination Survey V and VI (2010–2013) were used. Men and women with cardiovascular diseases showed high risks of stress, depression, and suicidal ideation. Men with metabolic diseases had worse mental health in relation to depression and suicidal ideation, and women with metabolic diseases in relation to stress and depression, indicating a need for intervention and management of mental health by gender for these individuals. There is a need to develop a clear perception and understanding, both among practitioners and the general population, of mental health issues associated with having cardiovascular or metabolic diseases. Active, specific health improvement and training programmes considering gender differences are necessary.

## 1. Introduction

Cardiovascular diseases (CVDs) are a main cause of death and disability and can be a major public health burden worldwide [1]. Therefore, primary medical care and prevention of CVDs are important, and this requires a comprehensive approach. For the management of CVD risk factors, lifestyle counselling, weight control, cholesterol-lowering medication, and blood pressure monitoring are necessary [2]. Furthermore, CVDs are related to a decrease in health-related quality of life, with different effects according to gender [3,4].

Metabolic diseases, which appear as a combination of abdominal obesity, high blood pressure, dyslipidaemia, and high blood sugar, in addition to insulin resistance, are seen as a high-risk factor for death [5,6]. Lifestyle habits are reported as an important factor in metabolic diseases, and stress and depression are also considered to be related [7]. Emotional stress is common in modern society, and the prevalence of metabolic diseases will continue to increase as life expectancy increases and lifestyles, including eating habits, become more Westernised [8]. Metabolic diseases and a high CVD risk is associated with impaired quality of life and a combination of biological and psychological risk factors [3,6,7,9].

Older adults with chronic diseases often experience disabilities in their daily lives because of a decrease in physical function, and the loss of social support creates a very unhealthy physical and mental state [10]. A reduction in physical function can be a causal factor of stress, depression, and suicide through a reduction in activity and psychological withdrawal due to difficulty with interpersonal relationships [11]. Physical illness can be a factor that induces depression and a factor that worsens physical problems when appropriate intervention for depression has not taken place [12,13]. Physical health problems, such as metabolic diseases and CVDs, can lower self-esteem as well as cause psychological or psychopathological problems such as depression, anxiety, stress, and suicidal impulses [14,15,16].

The prevalence and incidence rates of CVDs are increasing rapidly in Korea [4]. In metabolic diseases and CVD cases, the incidence rate and prognosis in women after menopause are worse than in men [17]. Women are known to have rapidly increased prevalence rates of cardiocerebrovascular diseases after the age of 40, owing to menopause-related female hormone changes, physical changes from ageing, and increased fat accumulation in the body [18]. Depression is also a major mental health problem in middle-aged Korean women. Changes in female hormones induce somatisation symptoms and depression, the rates of which are about twice as high as in men [8,19].

In previous studies, metabolic diseases and CVD were related to mental health including depression and stress [10,11,15], and also differed according to gender [3,16,17]. Therefore, this study confirms this by classifying each gender group and analyzing the differences in mental health between patients with CVDs or metabolic disease and general adults without these diseases for each group. Physical health problems, such as metabolic diseases and CVDs, affect mental health and the prognosis and incidence rates of these disorders show differences by age; studies comparing the mental health of adults with metabolic diseases or CVDs by gender and with the general population are insufficient. Therefore, in this study, we used raw data from the Korea National Health and Nutrition Examination Survey (KNHANES) V and VI (2010–2013), a large-scale, representative, and reliable investigation, to identify differences in the mental health between adults without these diseases and patients with CVDs or metabolic diseases in each gender.

## 2. Methods

### 2.1. Aims

The question this study attempts to answer is as follows. “Is there a difference in the mental health of patients with CVDs or metabolic disease and general adults without these diseases in each gender?

### 2.2. Design

This study employed a cross-sectional design.

### 2.3. Sample

The KNHANES used a stratified, multi-stage, clustered, probability sampling design to ensure the sample’s representativeness of all Korean citizens. Furthermore, the KNHANES V and VI use a rolling survey sampling method. The rolling sample in each survey year is a probability sample representative of the country as a whole and has independent and homogeneous characteristics between the samples. This study selected a total of 23,217 subjects (9828 men, 13,389 women) over 19 years of age excluding missing values from the KNHANES V and VI data (*n* = 33,551). Of the male subjects in our study’s sample, 566 (5.76%) had CVDs and 1389 (14.1%) had metabolic diseases. Among female subjects, 518 (3.87%) had CVDs and 1985 (14.8%) had metabolic diseases. In this study, adults without CVD and metabolic diseases were considered as general adults.

### 2.4. Data Collection

Since 1998, the Korea Centers for Disease Control and Prevention (KCDC) has conducted the KNHANES to identify the state of Korean health and nutrition. The KNHANES is a nationally representative, cross-sectional survey targeting non-institutionalised Korean people and comprises a health questionnaire, nutrition examination, and physical examination. The health questionnaire and nutrition examination are conducted through one-on-one interviews and self-reports while physical examinations are performed by the KCDC examination team.

### 2.5. Assessments

#### 2.5.1. CVDs

CVDs in this study signify stroke, myocardial infarction, and angina. We analysed those who had been diagnosed with stroke, myocardial infarction, or angina by a doctor through the health interview survey. Adults with both cardiovascular and metabolic diseases were included in the CVD group for analysis.

#### 2.5.2. Metabolic Diseases

Metabolic diseases in this study refer to diabetes mellitus and hyperlipidaemia. We analysed those who were diagnosed with diabetes mellitus or hyperlipidaemia by a doctor through the health interview survey.

#### 2.5.3. Mental Health

To assess the mental health of the subjects, we used the variables of stress, depression, and suicidal ideation. For stress, we categorised responses to the question ‘How much stress do you experience in regular day-to-day life?’ by defining the responses ‘I experience very high levels of stress’ and ‘I experience high levels of stress’ as ‘Yes’ and the responses ‘I tend to experience it a little bit’ and ‘I barely experience it’ as ‘No’. Depression was identified by a ‘Yes’ response to the question ‘Have you felt such sadness or despair during the previous year that your daily life has been disrupted for more than two weeks in a row?’ Suicidal ideation was identified by a ‘Yes’ response to the question ‘Did you ever think that you want to die during the past year?’

#### 2.5.4. Covariates

For the subjects’ demographic and health-related variables, we used age, education, marital status, living alone, body mass index (BMI), economic status, current employment, smoking, drinking, and physical activity. Education was categorised into below elementary school, middle school, high school, and college or above. Marital status was categorised into married and unmarried. Living alone was categorised based on whether the subject was then living alone. BMI was calculated using the equation weight(kg)/height(m)^2^. For economic status, the equivalent income (monthly average household income/$\sqrt{\mathrm{number}\;\mathrm{of}\;\mathrm{members}\;\mathrm{in}\;\mathrm{the}\;\mathrm{household}}$) that adjusts household income by the number of members in the household was categorised into quartiles. Employment status was determined by whether the subject was currently working. For smoking, subjects who smoked at the time of the survey were categorised as ‘Yes’ and others as ‘No’. Based on their drinking experience over the previous 12 months, subjects who drank alcohol more than once per month were categorised as ‘Yes’ and the rest as ‘No’. For physical activity, subjects who performed moderate-intensity physical activity for more than 2.5 h per week or high-intensity physical activity for more than one hour and 15 min or a mix of moderate and high-intensity physical activity were categorised as ‘Yes’ and others as ‘No’ [20,21].

### 2.6. Ethical Considerations

The KNHANES V and VI were conducted with approval from the Institutional Review Board of KCDC (IRB No. 2010-02CON-21-C, 2011-02CON-06-C, 2012-01EXP-01-2C, 2013-07CON-03-4C) and informed consent was obtained from each participant prior to the survey. Formal approval from the corresponding institution was obtained for the analysis of raw data.

### 2.7. Data Analysis

All data are presented as mean ± standard deviation for continuous variables or as n (%) for categorical variables. SAS version 9.3 (SAS Institute Inc., Cary, NC, USA) was used to run a complex sample design based on data analysis from the survey data; this provided sampling weights for the KNHANES and nationally representative estimates. The statistical significance of all results was tested based on *p* < 0.05.

The differences between the types of group (CVDs, metabolic diseases, and adults in general) regarding demographic characteristics, health-related characteristics, and mental health were determined using the χ^2^ test. To identify the association between the subjects’ group types and their mental health, the demographic and health-related characteristics that were significant in the simple difference test were adjusted as covariates and a logistic regression analysis was performed.

## 3. Results

### 3.1. Demographic and Health-Related Characteristics by Group according to Gender

The differences between those with CVDs, those with a metabolic disease, and the general group based on the demographic and health-related characteristics of men are presented in Table 1. Men showed significant differences by group for all characteristics.

Men with CVD were the oldest (*p* < 0.001), and the proportion with a low educational level was also high among men with CVD (*p* < 0.001). There were differences among the three groups in relation to marital status (*p* < 0.001), but most subjects were married. In all three groups, most did not live alone, but the proportion of those living alone was higher in men with CVD compared to those with metabolic diseases or men in general (*p* < 0.001). BMI was higher in men with CVDs or metabolic diseases compared to men in general (*p* < 0.001), and the proportion of those with very low economic status was higher in men with CVDs (*p* < 0.001). The proportion currently employed was low in men with CVDs (*p* < 0.001), as was the proportion of current smokers (*p* < 0.001). Although the proportion of current drinkers was low in men with CVDs, it was high in men with metabolic diseases (*p* < 0.001). The proportion of subjects who performed more than moderate-intensity physical activity was found to be low in men with CVDs (*p* = 0.019).

The differences between the CVD group, the metabolic diseases group, and the general group based on the demographic and health-related characteristics of women are presented in Table 2. Women showed significant differences by group for all characteristics except for physical activity.

Women with CVDs were the oldest, followed by women with metabolic diseases (*p* < 0.001). The proportion of subjects with a low educational level was higher in women with CVDs, followed by women with metabolic diseases (*p* < 0.001). Again, while there were differences in marital status between the three groups (*p* < 0.001), almost all subjects were married. Most did not live alone, but the proportion of those living alone was higher in the women with CVDs and those with metabolic diseases compared to women in general (*p* < 0.001). BMI was higher in women with CVDs or metabolic diseases compared with women in general (*p* < 0.001), and the proportion of very low economic status was higher in women with CVDs or metabolic diseases (*p* < 0.001). The proportion of those currently employed was low in women with CVDs or metabolic diseases (*p* < 0.001), as was the proportion of women with CVDs or metabolic diseases who were current smokers (*p* < 0.001). However, the proportion of current drinkers was found to be high in women with CVDs (*p* < 0.001).

### 3.2. Mental Health according to Types of Group by Gender

The differences in mental health among the CVD group, the metabolic diseases group, and the general group by gender are presented in Table 3. Men showed differences by disease in stress, depression, and suicidal ideation, while women showed differences by disease in depression and suicidal ideation only.

Men in general were found to experience more stress compared to those with CVDs or metabolic diseases (*p* = 0.002). The proportions of those with depression and suicidal ideation were the highest among men with CVDs, followed by men with metabolic diseases (*p* < 0.001). For women, the proportions for depression and suicidal ideation were the highest in those with CVDs, followed by those with metabolic diseases (*p* < 0.001).

Table 4 presents the association between mental health and diseases by gender. All demographic and health-related characteristics that were significant in the simple difference test were adjusted. In other words, age, education, living alone, marital status, economic status, BMI, occupation, smoking, drinking, and physical activity were adjusted for men, and age, education, living alone, marital status, economic status, BMI, occupation, smoking, and drinking were adjusted for women. In the final results, when comparing men with CVDs to men in general as reference, the odds ratio (OR) of stress was shown to be 1.34 (95% confidence interval (CI): 1.06 to 1.69), the OR of depression was 1.49 (95% CI: 1.13 to 1.97), and the OR of suicidal ideation was 1.67 (95% CI: 1.29 to 2.16). Furthermore, when comparing men with metabolic diseases to men in general as reference, the OR of depression was 1.47 (95% CI: 1.21 to 1.79) and the OR of suicidal ideation was 1.23 (95% CI: 1.01 to 1.50).

When comparing women with CVDs to women in general as reference, the OR of stress was 1.31 (95% CI: 1.07 to 1.61), the OR of depression was 1.44 (95% CI: 1.15 to 1.79), and the OR of suicidal ideation was 1.53 (95% CI: 1.23 to 1.90). In addition, when comparing women with metabolic diseases to women in general as reference, the OR of stress was found to be 1.20 (95% CI: 1.06 to 1.35), and the OR of depression was 1.26 (95% CI: 1.10 to 1.44).

## 4. Discussion

In this study, we compared the mental health of adults with CVDs or metabolic diseases with adults without these disease in each gender. Through this, we contributed to the expansion of understanding of adults with CVDs or metabolic diseases and identified a need for intervention to maintain and improve mental health depending on gender and the specific disease group.

According to the results, men with CVDs or metabolic diseases showed differences depending on age, education, living alone, marital status, economic status, BMI, current employment, smoking, drinking, and physical activity, while women showed differences depending on age, education, living alone, marital status, economic status, BMI, current employment, smoking, and drinking. For men, those with CVDs were older and had a lower socioeconomic status, but for women, this was true for both the group with CVDs and that with metabolic diseases. Furthermore, the BMI of adults with CVDs and metabolic diseases was high for both men and women. The proportion of those adults not smoking but still drinking alcohol was high, and in particular, the proportion of subjects who performed moderate-intensity physical activity was low among men with CVDs or metabolic diseases. In previous studies [5,7,22,23,24], differences in age, economic status, educational level, and gender were highlighted. Women over 50 years old experience menopause and show a tendency toward a rapidly increased prevalence of metabolic diseases due to the lack of oestrogen [4]. In addition, more health-related problems in women are reported after middle age, with the overall prevalence rate of diseases other than CVDs being higher in women than in men [6,14]. Moreover, women have lifestyle habits (such as irregular meals, exercise, and diet) that are different from men [24]. The need for customised programmes to suit individual situations that take into consideration the characteristics and risks of middle-aged women who are less physically active owing to lack of time given low income, non-regular work, housework, and child care is emphasised [19,25]. Therefore, this study shows that education and intervention based on considerations of differences in gender and disease are necessary for healthy living habits.

The final results show that both male and female adults with CVDs had high stress and depression levels, and the risk of suicidal ideation was also found to be high. Men with metabolic diseases had high risks of depression and suicidal ideation, while women with metabolic diseases experienced more stress and depression compared to women in general. Therefore, it was seen that adults with CVDs or metabolic diseases had worse mental health compared with adults in general. It was also found that adults with CVDs had worse mental health than adults with metabolic diseases.

Iftikhar et al. [26]. reported that psychological factors such as stress, depression, suicidal ideation, and sleep duration were associated with metabolic diseases. Metabolic diseases can be a result of psychosocial problems, such as stress and bad lifestyle habits [15]. Therefore, lifestyle habits and psychosocial factors are seen to have interactive relationships in both metabolic diseases and CVDs. The finding that visceral fat, which contributes to central adiposity, is related to depression [14] also supports the results of our study.

The ability to adapt to chronic stress differs depending on gender and is affected by sex hormones [27]. Furthermore, high-stress environments can induce the accumulation of organ fat through the activation-centred pathway of stimulating the adrenal gland that releases glucocorticoids, leading to chronic hypercortisolism [28]. Therefore, subjects who experience more stress are seen to have an increased risk of metabolic diseases compared to subjects who experience less stress [23].

Metabolic diseases in patients with coronary heart disease has been associated with psychological risk factors (depression or high levels of anger or hostility) [7,29] as well as depression and CVDs [22,30]. Additionally, stress over the condition of their general health and its possible deterioration can increase the risk of depression in patients with CVDs [30].

Depression is a significant risk factor for poor mental health; it induces sleep disorder and a reduction in activity, which then creates a vicious cycle of further worsening stress and depression [13,24,26,31]. Given that depression is associated with the occurrence of CVDs and that depression is a factor that delays and prevents proper treatment of CVDs [19], it is believed that active management of mental health is critical in the treatment of CVDs. In the United States, it is actually recommended that patients hospitalised for CVD be checked for depression; this is also included in recommendations for follow-up care [32]. In light of the findings that high blood pressure, angina, and diabetes did not affect depression and stress but that both depression and stress were affected in cases of serious obesity [16], an intervention programme that improves lifestyle habits rather than focusing on symptom alleviation may be necessary.

Previous studies consistently report that depression is closely associated with suicide attempts and suicide itself [13,33]. The current study suggests that higher physical activity levels are associated with lower suicidal ideation [34]. Physical activity is associated with numerous health benefits, including enhanced emotional and physical health, improved cognitive function, improved sleep and better quality of life [9,35]. Physical activity may be inversely related to SI as a result of neurobiological alterations that occur with physical activity; metabolic disease and CVDs also can be improved through physical activity. Thus, the present study suggests the need for active intervention in relation to mental health as well as intervention for symptoms of metabolic syndrome or CVDs.

Psychological and psychosocial factors such as depression and stress can also cause metabolic disease [8,14]. The relationship between socioeconomic background and metabolic disease has been reported in previous studies [10,12,14]. Efforts to proactively identify and respond to modifiable risk factors for metabolic disease may serve as factors that prevent metabolic disorders and improve quality of life.

The results indicate the need to develop a clear perception and understanding, both among practitioners and the more general population of the mental health issues associated with having CVDs or a metabolic disease. Additionally, active and specific health improvement and training programs that take into account differences not only in the diseases but also between genders are necessary in the future. Furthermore, a variety of actions need to be taken to improve the mental health of adults with CVDs or metabolic disease; in the case of adults with CVDs, the need is especially urgent.

Postmenopausal depression or stress decreases physical activity, resulting in increased metabolic disease and CVDs [24]. In cases of women with low socioeconomic levels, since they have housework and jobs at the same time, metabolic disease increases due to depression resulting from stress and economic deprivation [4,10]. Metabolic disease shows different patterns depending on gender and socioeconomic status [4,26]. Therefore, it is necessary to establish a differentiated strategy for the prevention of metabolic diseases and CVDs and intervention that considers mental health, diet, physical activity, and socioeconomic level for each male and female group. This study categorised the mental health of adults with CVDs or metabolic diseases by gender and compared them to the general adult population. One of the major strengths of this study is its large size and the representativeness of the sample used in the analysis. Despite this, the study does suffer from some limitations. First, because information about the severity of CVDs or metabolic diseases is not available, we could not investigate the relationship between the severity of these conditions and mental health. Furthermore, metabolic diseases in our study only included diabetes mellitus and hyperlipidaemia. Future research should, therefore, broaden the scope by including other metabolic diseases. Moreover, because this is a cross-sectional study, causal relationships could not be deciphered; this suggests the need for a longitudinal study in the future. Despite these limitations, our study is significant in that it is the first to examine the mental health of Korean adults with CVDs or metabolic diseases by gender and compare them to the general adult population.

## 5. Conclusions

In summary, our study found that adults with CVDs or metabolic diseases had worse mental health than the general population, that this was especially true for those with CVDs, and that the nature of the mental health effects varied by gender. Based on the findings of our study, the development of a multidisciplinary intervention programme for the improvement of mental health in patients with metabolic diseases and CVDs is urgently needed. There is also a need for more attention and research into efforts to improve the mental health of such patients.

## Figures and Tables

**Table 1:** Demographic and health-related characteristics of men.

Characteristic		Total (*N* = 9828) Mean ± SD or *n* (%)	CVDs (*n* = 566) Mean ± SD or *n* (%)	Meta (*n* = 1389) Mean ± SD or *n* (%)	General (*n* = 7873) Mean ± SD or *n* (%)	*p*-Value
Age (years)		50.4 (16.7)	65.9 (10.5)	59.5 (11.9)	47.9 (16.3)	<0.001
Education	≤Elementary school	1704 (17.4)	184 (32.5)	330 (23.8)	1190 (15.1)	<0.001
	Middle school	1140 (11.6)	103 (18.2)	233 (16.8)	804 (10.2)	
	High school	3560 (36.2)	171 (30.2)	448 (32.2)	2941 (37.4)	
	≥College	3424 (34.8)	108 (19.1)	378 (27.2)	2938 (37.3)	
Marital status	Married	9243 (83.3)	554 (97.7)	1342 (96.5)	7347 (80.4)	<0.001
	Unmarried	1846 (16.7)	13 (2.3)	48 (3.5)	1785 (19.6)	
Living alone	Yes	562 (5.1)	49 (8.6)	75 (5.4)	438 (4.8)	<0.001
BMI		23.7 (3.4)	24.2 (2.9)	24.6 (3.0)	23.9 (3.2)	<0.001
Economic status	Very low	1981 (18.0)	212(37.7)	309(22.4)	1460(16.2)	<0.001
	Low	2856 (26.0)	121 (21.5)	356 (25.8)	2379 (26.3)	
	High	3033 (27.6)	114 (20.2)	350 (25.3)	2569 (28.4)	
	Very high	3111 (28.3)	116 (20.6)	366 (26.5)	2.6269 (29.1)	
Employment (current)	Yes	7286 (74.1)	259 (45.8)	926(66.0)	6.111 (77.6)	<0.001
Smoking (current)	Yes	5172 (46.5)	150 (26.4)	489 (35.2)	4533 (49.5)	<0.001
Drinking (current)	Yes	7774 (69.9)	357 (62.9)	1043 (75.0)	6374 (69.6)	<0.001
Physical activity (moderate)	Yes	874 (8.9)	33 (5.9)	115 (8.3)	726 (9.2)	0.019

Notes. CVDs: cardiovascular diseases, meta: metabolic diseases, BMI: body mass index.

**Table 2:** Demographic and health-related characteristics of women.

Characteristic		Total (*N* = 13,389) Mean ± SD or *n* (%)	CVDs (*n* = 518) Mean ± SD or *n* (%)	Meta (*n* = 1985) Mean ± SD or *n* (%)	General (*n* = 10,886) Mean ± SD or *n* (%)	*p*-Value
Age (years)	50.35		67.35	63.08	47.55	<0.001
	(16.7)		(9.4)	(10.8)	(16.67)	
Education	≤Elementary school	4172	378	1095	2699	<0.001
		(31.2)	(73.0)	(55.2)	(24.8)	
	Middle school	1356	69	302	985	
		(10.1)	(13.3)	(15.2)	(9.0)	
	High school	4255	58	432	3765	
		(31.8)	(11.2)	(21.8)	(34.6)	
	≥College	3606	13	156	3437	
		(26.9)	(2.5)	(7.8)	(31.6)	
Marital status	Married	12,675	519	19,656	10,200	<0.001
		(87.1)	(99.6)	(98.5)	(84.7)	
	Unmarried	1882	2	30	1850	
		(12.9)	(0.4)	(1.5)	(15.3)	
Living alone	Yes	1342	108	332	902	<0.001
		(9.2)	(20.7)	(16.7)	(7.5)	
BMI	23.66		25.11	24.87	23.07	<0.001
	(3.4)		(3.3)	(3.5)	(3.5)	
Economic status	Very low	3059	239	662	2158	<0.001
		(21.3)	(46.3)	(33.7)	(18.0)	
	Low	3768	136	563	3069	
		(26.2)	(26.4)	(28.7)	(25.8)	
	High	3735	85	368	3282	
		(26.0)	(16.5)	(16.8)	(27.6)	
	Very high	3826	56	369	3401	
		(26.5)	(10.8)	(18.8	(28.6)	
Employment (current)	Yes	6338	136	661	5541	<0.001
		(47.3)	(26.3)	(33.3)	(50.1)	
Smoking (current)	Yes	1697	18	75	1604	<0.001
		(11.6)	(3.5)	(3.8)	(13.3)	
Drinking (current)	Yes	8007	327	1124	6556	<0.001
		(54.9)	(62.8)	(56.5)	(54.3)	
Physical activity (moderate)	Yes	966	28	144	794	0.288
		(7.2)	(5.5)	(7.3)	(7.3)	

Notes. CVDs: cardiovascular diseases, meta: metabolic disease, BMI: body mass index.

**Table 3:** Differences in mental health by group according to gender.

Mental Health	Men (*n* = 9828)				Women (*n* = 13,389)			
	CVDs (*n* = 566)	Meta (*n* = 1389)	General (*n* = 7873)	*p*-Value	CVDs (*n* = 518)	Meta (*n* = 1985)	General (*n* = 10,886)	*p*-Value
	*n* (%)	*n* (%)	*n* (%)		*n* (%)	*n* (%)	*n* (%)	
Stress	105	276	1832	0.002	156	546	3020	0.46
	(18.7)	(19.9)	(23.3)		(30.2)	(27.6)	(27.8)	
Depression	75	163	617	<0.001	126	397	1629	<0.001
	(13.3)	(11.8)	(7.8)		(24.4)	(20.1)	(15.0)	
Suicidal ideation	93	152	618	<0.001	136	362	1527	<0.001
	(16.5)	(10.1)	(7.8)		(26.4)	(18.3)	(14.0)	

Notes. CVDs: cardiovascular diseases, meta: metabolic disease.

**Table 4:** Association between mental health and group by gender.

Gender		Stress OR (95% CI)	Depression OR (95% CI)	Suicidal Ideation OR (95% CI)
Men	Adjusted (age, education, living alone, marital status, economic status, BMI, current employment, smoking, drinking, physical activity)			
	CVDs	1.34	1.49	1.67
		(1.06, 1.69)	(1.13, 1.97)	(1.29, 2.16)
	Meta	1.12	1.47	1.23
		(0.96, 1.31)	(1.21, 1.79)	(1.01, 1.50)
	General	1	1	1
Women	Adjusted (age, education, living alone, marital status, economic status, BMI, current employment, smoking, drinking)			
	CVDs	1.31	1.44	1.53
		(1.07, 1.61)	(1.15, 1.79)	(1.23, 1.90)
	Meta	1.2	1.26	1.1
		(1.06, 1.35)	(1.10, 1.44)	(0.96, 1.26)
	General	1	1	1

Notes. OR: odds ratio, CI: confidence interval, CVDs: cardiovascular diseases, meta: metabolic disease, BMI: body mass index.
